# MQAPRank: improved global protein model quality assessment by learning-to-rank

**DOI:** 10.1186/s12859-017-1691-z

**Published:** 2017-05-25

**Authors:** Xiaoyang Jing, Qiwen Dong

**Affiliations:** 1 School of Computer Science, Fudan University, Shanghai, 200433 People’s Republic of China; 2 School of Data Science and Engineering, East China Normal University, Shanghai, 200062 People’s Republic of China

**Keywords:** Protein structure prediction, Protein model quality assessment, Learning-to-rank

## Abstract

**Background:**

Protein structure prediction has achieved a lot of progress during the last few decades and a greater number of models for a certain sequence can be predicted. Consequently, assessing the qualities of predicted protein models in perspective is one of the key components of successful protein structure prediction. Over the past years, a number of methods have been developed to address this issue, which could be roughly divided into three categories: single methods, quasi-single methods and clustering (or consensus) methods. Although these methods achieve much success at different levels, accurate protein model quality assessment is still an open problem.

**Results:**

Here, we present the MQAPRank, a global protein model quality assessment program based on learning-to-rank. The MQAPRank first sorts the decoy models by using single method based on learning-to-rank algorithm to indicate their relative qualities for the target protein. And then it takes the first five models as references to predict the qualities of other models by using average GDT_TS scores between reference models and other models. Benchmarked on CASP11 and 3DRobot datasets, the MQAPRank achieved better performances than other leading protein model quality assessment methods. Recently, the MQAPRank participated in the CASP12 under the group name FDUBio and achieved the state-of-the-art performances.

**Conclusions:**

The MQAPRank provides a convenient and powerful tool for protein model quality assessment with the state-of-the-art performances, it is useful for protein structure prediction and model quality assessment usages.

## Background

In the last two decades, various protein three-dimensional structure prediction methods have been developed and much progress has been made in this area [1]. Generally, numerous predicted decoy models are generated for a given protein sequence, and correctly ranking these models and selecting the best predicted model from the candidate pool remain challenging tasks. Over the past years, a number of methods have been developed to address this issue [2, 3], and these methods could roughly be divided into three categories: single methods, quasi-single methods and clustering (or consensus) methods. The single methods evaluate the model quality using the inputted model only [4–6] and often use three conceptual approaches: the physical model, the statistical model and the comparison between predicted properties and the properties extracted from decoy models. The quasi-single methods identify a few high-quality models as references, and evaluate the subsequent models by comparing them with the reference models [7, 8]. The clustering methods often use clustering algorithm to cluster a set of models generated by structure prediction programs for target sequence [9–12]. The clustering methods generally outperform single-model methods when numerous models are available [13, 14], however, the clustering methods perform poorly if most models are of low qualities or only a few models are available.

In this work, we developed a novel program based on learning-to-rank for protein model quality assessment (MQAPRank). First, the MQAPRank formulates the protein model quality assessment task as a ranking task and uses single method to sort the decoy models, the features include knowledge-based mean force potentials and evaluation scores from other state-of-the-art MQAP (model quality assessment program). Then, the MQAPRank takes the first five decoy models ranked by the learning-to-rank algorithm as the reference models and the predicted qualities of other models are the average GDT_TS score of the target models with the five reference models. The MQAPRank has been evaluated on the CASP11 (11th Community Wide Experiment on the Critical Assessment of Techniques for Protein Structure Prediction) dataset and participated in the CASP12 (12th Community Wide Experiment on the Critical Assessment of Techniques for Protein Structure Prediction) recently, it achieves the state-of-the-art performances on those two datasets.

## Implementation

### Overview

**Fig. 1:**
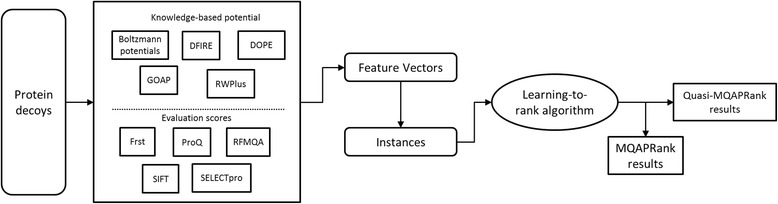
The overall flowchart of the proposed MQAPRank

The MQAPRank formulates the model quality assessment of protein models as a ranking problem, and the protein decoy models are sorted by their similarities with the corresponding native structures. Such similarities can be measured by various structure comparison programs and in the MQAPRank the GDT_TS score is adopted. The assessment procedure of MQAPRank consists of three steps and its overall flowchart is shown in Fig. 1. First, the MQAPRank extracts two kinds of features from the decoy models: knowledge-based mean force potentials and the evaluation scores of several programs for protein model quality assessment. The knowledge-based potentials used in the MQAPRank include Boltzmann-based potentials, DFIRE potential, DOPE potential, GOAP potential and RWplus potential. The evaluation scores from other protein model quality assessment programs include Frst, ProQ, RFMQA, SIFT and SELECTpro software, detailed descriptions of those features are shown in the features section. Then, each decoy model is represented as a feature vector and a pair of feature vector from the same protein is represented as an instance. These instances are inputted into learning-to-rank algorithm to predict the relative ranking relation of any two models from the same protein. Finally, the MQAPRank takes the first five models as the reference models and the predicted qualities of other models are the average GDT_TS score of the target models with the reference models.

In summary, the MQAPRank uses various features to predict the ranking relation of protein decoy models based on learning-to-rank algorithm and chooses first five best decoy models as references to score other decoy models. In order to provide more valuable assessment information, the MQAPRank will output both the initial learning-to-rank based score (MQAPRank score) and the final predicted score (quasi-MQAPRank score).

### Learning-to-rank algorithm

Learning-to-rank is a machine learning algorithm which constructs a ranking strategy and sorts new objects according to their relevance or importance to the target object. Learning-to-rank has been applied effectively to solve information retrieval problems, such as document retrieval, collaborative filtering, spam detection, etc. The existing learning-to-rank algorithms can be categorized into three approaches: pointwise approach, pairwise approach, and listwise approach, and different approaches model the process of learning-to-rank in different ways. The pairwise approach could apply existing methodologies on regression and classification and generally outperforms pointwise approach, thus we adopt the pairwise via-classification approach (SVMrank [15]) to deal with the protein model quality assessment problem. Specifically, the pairwise approach takes pairs of decoy models (represented as feature vectors) as instances for learning, and formalizes the task of ranking decoy models as that of classification. In learning, it first collects decoy model pairs from the decoy model list of a certain protein, and then assigns a label representing the relative qualities of the two decoy models for each pair. The final process is to train a classification model with the labeled data and to make use of the model to rank new decoy models.

### Features

The MQAPRank extracts two kinds of features from the decoy models: knowledge-based mean force potentials and the evaluation scores of several programs for protein model quality assessment.

#### Knowledge-based potentials

The knowledge-based potentials include Boltzmann-based potentials [16], DFIRE potential [17], DOPE potential [18], GOAP potential [19] and RWplus potential [20].

The Boltzmann-based potentials are widely used mean force potentials that is derived from the inverse Boltzmann law, and the corresponding non-linear forms are proposed in our previous study [16]. The five Boltzmann-based potentials include the DIH potential [21], the DFIRE-SCM potential [22], FS potential [23], HRSC potential [24], T32S3 potential [25].

The DFIRE potential [17] is a distance-dependent structure-derived potential, which sums the interactions of all pairs of non-hydrogen atoms (167 atomic types).

The DOPE (Discrete Optimized Protein Energy) potential [18] is based on an improved physical reference state that corresponds to non-interacting atoms in a homogeneous sphere with the radius dependent on a sample native structure. Its variants (DOPE-normal (Normalized DOPE by z score) and DOPE-HR (the bin size is 0.125 Å, a higher resolution than DOPE)) are also used in the MQAPRank.

The GOAP potential [19] is a generalized orientation and distance-dependent all-atom statistical potential, which depends on the relative orientation of the planes associated with each heavy atom in interacting pairs.

The RWplus potential [20] is based on the pair-wise distance-dependent atomic statistical potential function RW [26], and contains a side-chain orientation-dependent energy term.

#### Evaluation scores from other MQAPs

The evaluation scores from other model quality assessment programs are also extracted as additional features, which include the Frst [27], ProQ [5], RFMQA [28], SIFT [29] and SELECTpro [30].

The output of the Frst [27] is based on four knowledge-based potentials: RAPDF potential, SOLV potential, HYDB potential, and TORS potential, and the Frst energy is a weighted linear combination of the four potentials. Besides the combination potential, the individual potentials are also used as the features in the MQAPRank.

The ProQ [5] is a neural-network-based method to predict the protein model quality. It uses structural information which contains the frequency of atom contacts and residue contacts, solvent accessibility surfaces, the fraction of similarity between predicted secondary structure and the secondary structure in the model, and the difference between the all-atom model and the aligned C-alpha coordinates from the template.

The RFMQA [28] is a random forest based model quality assessment using structural features and knowledge-based potential energy terms. Here we used an analogous strategy as RFMQA to extract four protein secondary structure features and two solvent accessibility features. For protein secondary structure features, the focus is the consistency between predicted and actual secondary structures of a target protein. For each decoy model, we use DSSP [31] to calculate its secondary structures and PSIPRED [32] to predict the secondary structures of the target sequence. The fraction of consistent secondary structural element (alpha-helix, beta-strand and coil) between the DSSP label and the PSIPRED output is calculated by dividing the consistency number by its total chain length, and the total consistency RFMQA-SS-total score is also used as a feature. For solvent accessibility features, the absolute solvent accessibility of the model is computed by DSSP and relative solvent accessibility is computed by ACCpro5 [33]. These two vectors are compared and transformed into a Pearson Correlation Coefficient and a cosine value as two features.

The SIFT [29] is a program which uses averaged (i.e. amino acid independent) radial distribution functions (RDF) to discriminate properly packed models from misfolded ones. It produces two alternative scores: one based on RDF only and the other based on a combination of RDF and other sequence-independent filters.

The SELECTpro [30] is a structure-based model assessment method derived from an energy function comprising physical, statistical, and predicted structural terms that include predicted secondary structure, predicted solvent accessibility, predicted contact map, β-strand pairing and side-chain hydrogen bonding.

### Usage

#### Web Server

We offer a web server to non-commercial users at http://dase.ecnu.edu.cn/qwdong/MQAPRankWebServer/server. Non-commercial users could upload decoy models of protein targets to the server and get predicted GDT_TS values of corresponding models by the learning-to-rank (MQAPRank score) and the predicted GDT_TS value by the quasi-clustering method (quasi-MQAPRank score) from the result page.

#### Stand-alone Program

The standalone program of MQAPRank is implemented in Python 2.7.6. The source code, installation tutorial and test example are freely available to non-commercial users at http://dase.ecnu.edu.cn/qwdong/MQAPRankWebServer/software. To reduce the complexity of the usage, the MQAPRank uses one call script to execute the task. The input of MQAPRank is a text file which contains the full path of protein models to be evaluated. Users could chose the structure similarity metric (GDT_TS or TMscore) to be used by MQAPRank. The output is a text file that contains three items in every line: full path of the model, the predicted value of the corresponding model by the learning-to-rank (MQAPRank score) and the predicted value of the corresponding model by the quasi-clustering method (quasi-MQAPRank score).

## Results and discussion

### Performance comparison on CASP12 dataset

**Fig. 2:**
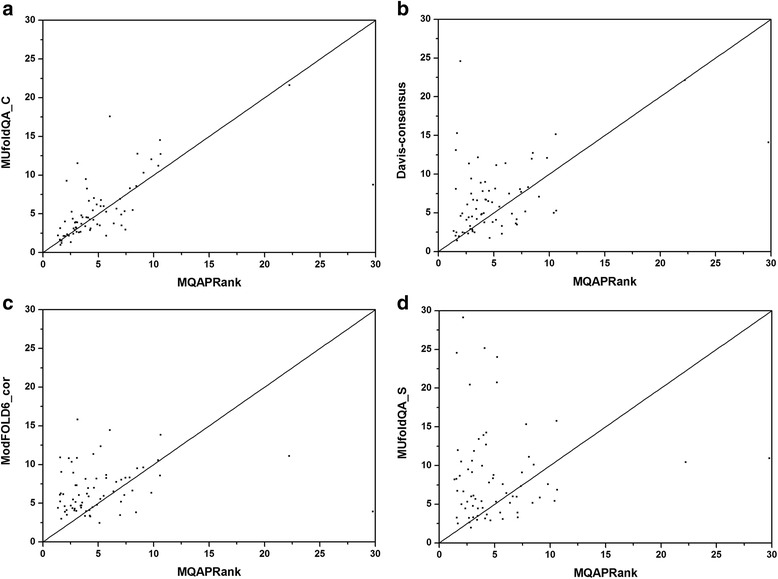
Comparison of the performance on Diff metric between the MQAPRank and other methods. **a** MUfoldQA_C. **b** Davis-consensus. **c** ModFOLD6_cor. **d** MUfoldQA_S. (Line x = y is shown for reference. Due to smaller Diff value indicates better performance, the method with less *scatter points* is better in this figure.)

The MQAPRank has participated in the CASP12 under the group name FDUBio. Its performances and corresponding performances of four selected methods in CASP12 are shown in Table 1. All of the performances on the CASP12 dataset are obtained from the CASP12 official website (http://www.predictioncenter.org/casp12), three of the four selected methods are leading methods with best performances in their corresponding categories based on the Diff metric. Specifically, the MUfoldQA_C is the leading method in clustering category, the ModFOLD6_cor and MUfoldQA_S are the leading methods in quasi-single category and single category respectively. The Davis-consensus is the reference clustering method for assessing progress in protein model quality assessment field. On the CASP12 dataset, compared with three leading methods and the reference method Davis-consensus, the MQAPRank outperforms other leading methods on all metrics on the best 150 dataset and achieves comparable performances on the select 20 dataset. Fig. 2 shows scatter plots of the Diff metric comparison between the MQAPRank and other four methods. It should be noted that smaller Diff value indicates better performance, so the method with less scatter points is better. As shown in the figure, most of decoy model qualities are better predicted by the MQAPRank.

**Table 1:** The performances of the MQAPRank and several leading methods on CASP12 dataset based on GDT_TS score

Method	Method Type	Best 150^a^				Sel20^b^			
		Diff^c^↓	MCC^d^↑	AUC^e^↑	Loss^f^↓	Diff↓	MCC↑	AUC↑	Loss↓
**MQAPRank**	quasi-clustering	**5.17**	**0.87**	**0.98**	**6.91**	5.76	0.41	0.93	7.18
MUfoldQA_C	clustering	5.51	0.84	**0.98**	7.46	3.82	0.15	0.96	**0.82**
Davis-consensus	clustering	6.78	0.83	**0.98**	7.68	5.61	0.00	0.78	15.56
ModFOLD6_cor	quasi-single	6.75	0.86	**0.98**	10.55	6.70	**0.86**	**0.99**	1.28
MUfoldQA_S	single	8.90	0.71	0.93	13.15	**3.60**	0.76	0.98	2.56

^a^Best 150: the dataset comprised of the best 150 models submitted on a target according to the benchmark consensus method. ^b^Select 20: the dataset comprised of 20 models spanning the whole range of server model difficulty on each target. ^c^Diff: The average difference between the predicted and GDT_TS scores. ^d^MCC: Matthews correlation coefficient (the threshold is 50 GDT_TS). ^e^AUC: The area under the ROC curve. ^f^Loss: The loss in quality between the best available model and the predicted best model. Bold value indicates highest performance

Three factors contribute to the success of the MQAPRank. The first one is the learning-to-rank framework which can give reasonable ranking of protein decoy models for a protein target. The MQAPRank formulates the protein model quality assessment problem as a ranking problem and sorts protein decoy models by their similarities with the corresponding native structures. The second one is the features which are the complementary outputs of various methods. These features reflect qualities of protein decoy models from different aspects, so the ranking could be more reasonable and comprehensive. The third one is the quasi-clustering (or quasi-single) strategy. The MQAPRank selects reference models based single method, which could avoid the typical shortcoming of clustering method and reduce the dependency of the distribution of decoy model qualities. In order to specifically demonstrate the success of MQAPRank, we select the protein target T0912 from CASP12 best 150 dataset as an example. The T0912 protein target is a long sequence protein with 624 residues and contains three domains, its tertiary structure is relatively hard to predict. The top 15 decoy models based on GDT_TS score are shown in Table 2, and the top five scored by GDT_TS and five methods are highlighted in bold. From the Table 2, we can see that four out of the first five decoy models ranked by the MQAPRank are consistent with those ranked by the GDT_TS score. The other decoy models predicted by the MQAPRank have quite similar scores with those scored by GDT_TS score. The MQAPRank successfully identifies high-quality decoy models from decoy model pool by using the learning-to-rank framework and complementary features, and then it takes the first five decoy models as references to reasonably score other models.

**Table 2:** The GDT_TS scores and predicted scores from different methods for the first 15 decoy models of target T0912 on best 150 dataset

Decoy model	GDT_TS	MQAPRank	MUfoldQA_C	Davis-consensus	ModFOLD6_cor	MUfoldQA_S
T0912TS005_1	**46.74**	**57.49**	32.58	25.02	32.24	34.87
T0912TS220_1	**45.47**	**57.19**	34.96	26.41	34.37	36.12
T0912TS005_3	**45.15**	**55.80**	33.20	25.49	32.75	35.14
T0912TS005_4	**44.83**	**56.63**	31.95	24.55	32.00	34.32
T0912TS479_1	**44.15**	43.79	**39.92**	**29.87**	**36.60**	**42.58**
T0912TS005_5	43.85	47.40	33.32	25.56	33.01	35.65
T0912TS005_2	43.77	**53.51**	32.24	24.88	32.63	34.62
T0912TS479_4	42.47	42.06	**38.18**	**28.47**	**34.80**	**41.02**
T0912TS183_4	41.31	41.76	**38.53**	**28.62**	**36.13**	**41.19**
T0912TS287_1	40.79	41.63	37.45	**28.41**	**35.20**	38.94
T0912TS357_2	40.63	40.36	**38.03**	28.28	33.78	**39.15**
T0912TS236_1	40.63	41.52	37.47	**28.43**	**35.05**	38.99
T0912TS220_2	40.38	40.31	32.43	24.82	33.17	34.19
T0912TS357_3	40.10	39.83	37.91	28.20	33.79	**39.15**
T0912TS357_1	39.94	39.93	**37.94**	28.19	33.59	39.05

Bold value indicates the first five decoy models withhighest GDT_TS score

### Performance comparison on CASP11 dataset

We have performed a benchmark evaluation on the CASP11 dataset to verify the ability of MQAPRank [34]. Referencing to the strategy of CASP [35], we use CASP10 dataset as the training set and make tests on the CASP11 dataset (Best 150 dataset and Select 20 dataset). We select four leading groups (Pcons-net, MULTICOM-CLUSTER, MULTICOM-REFINE and MQAPsingleA) from different categories and the CASP official reference method (DAVIS-QAconsensus) as references. Among these methods, the Pcons-net, MULTICOM-REFINE and DAVIS-QAconsensus are clustering methods, the MULTICOM-CLUSTER is a single method and the MQAPsingleA is a quasi-single method. We downloaded the performances of these four methods from the CASP11 official website (http://www.predictioncenter.org/casp11/index.cgi) and evaluated them by using metrics used in CASP12 and two more Pearson’s correlation coefficients between the predicted and GDT_TS scores. The evaluation results are shown in Table 3. As shown in the table, the MQAPRank achieves the state-of-the-art performances on the CASP11 dataset. These results are similar with those on the CASP12 dataset, which demonstrates the robustness of the MQAPRank.

**Table 3:** The performances of the MQAPRank and several leading methods on CASP11 dataset based on GDT_TS score

Method	Method Type	Best 150						Sel20					
		Diff	MCC	AUC	Loss	mPCC^a^	PCC^b^	Diff	MCC	AUC	Loss	mPCC	PCC
**MQAPRank**	quasi-clustering	**5.78**	**0.87**	**0.98**	**4.32**	**0.74**	**0.95**	**6.47**	**0.78**	0.97	9.55	0.77	0.91
MULTICOM-REFINE	clustering	6.06	0.87	**0.98**	7.62	0.68	0.94	7.99	0.61	**0.98**	5.20	0.90	0.92
DAVIS-QAconsensus	clustering	6.17	0.87	0.98	7.74	0.68	0.94	7.33	0.62	**0.98**	5.51	0.90	**0.95**
Pcons-net	clustering	7.50	0.81	0.98	5.28	0.71	0.94	9.08	0.57	**0.98**	**2.79**	0.91	0.93
MULTICOM-CLUSTER	single	13.2	0.66	0.91	7.06	0.43	0.79	12.4	0.62	0.92	9.47	0.71	0.82
MQAPsingleA	quasi-single	13.8	0.60	0.90	8.95	0.65	0.75	9.66	0.68	0.95	3.64	**0.92**	0.88

^a^mPCC: mean Pearson’s correlation coefficient between the predicted and GDT_TS scores of per target protein

^b^PCC: Pearson’s correlation coefficient between the predicted and GDT_TS scores on overall models. Bold value indicates highest performance on corresponding evaluation metric

### Performance comparison on 3DRobot dataset

We also evaluated the MQAPRank on a large dataset, 3DRobot dataset. The decoy models of 3DRobot are generated by the 3DRobot [36], a program devoted for automated generation of diverse and well-packed protein structure decoys. The 3DRobot dataset contains structural decoy models of 200 non-homologous proteins comprising by 48 α, 40 β, and 112 α/β single-domain proteins and the length of these proteins ranges from 80 residues to 250 residues. Each protein has 300 structural decoys with RMSD ranging from 0 Å to 12 Å, so there are 60000 decoy models in the 3DRobot dataset. We performed a benchmark evaluation of the MQAPRank on this dataset by using the five-fold cross-validation. We select one part (decoy models of 40 targets) as the test dataset and the remaining four parts (decoy models of 160 targets) as the train dataset each time. This process repeats five times and the prediction results of five test parts are integrated together finally.

In the meantime, we assessed decoy model qualities of the 3DRobot dataset by using three stand-alone programs (RFMQA [28], ModFOLDclust2 [37] and Pcons [14]) as references. The evaluation results are shown in Table 4. Table 4 shows that the MQAPRank outperforms other three methods, especially on the Diff metric. Compared with CASP datasets, the 3DRobot dataset contains much more decoy models for each protein target, and the distributions of decoy model qualities in it are more uniform. Due to these factors, the clustering methods (ModFOLDclust2 and Pcons), which are based on majority voting strategy, could not achieve ideal performances. While the MQAPRank still performs well by using learning-to-rank and quasi-clustering strategy.

**Table 4:** The performances of the MQAPRank on 3DRobot dataset based on GDT_TS score

Method	Method Type	Diff	MCC	AUC	Loss	mPCC	PCC
**MQAPRank**	quasi-clustering	**0.68**	**0.98**	**0.99**	**0.80**	**0.99**	**0.99**
RFMQA	single	9.73	0.74	0.96	1.70	0.92	0.87
ModFOLDclust2	clustering	11.42	0.80	**0.99**	7.51	0.95	0.90
Pcons	clustering	25.12	0.17	**0.99**	5.19	0.96	0.90

Bold value indicates highest performance on correspondingevaluation metric

## Conclusions

Assessing the qualities of protein decoy models in perspective is one of the key stages of protein structure prediction, but it is still an open problem. Here we propose the MQAPRank, which is a global protein model quality assessment program based on learning-to-rank, for protein structure prediction and protein model quality assessment usages. The evaluation results on the CASP12, CASP11 and 3DRobot datasets show that the MQAPRank could provide the state-of-the-art performance and is available for protein structure evaluation.

## Abbreviations

MQAP: Model quality assessment program
Best 150: The dataset comprised of the best 150 models submitted on a target according to the benchmark consensus method
Select 20: The dataset comprised of 20 models spanning the whole range of server model difficulty on each target
Diff: The average difference between the predicted and GDT_TS scores
MCC: Matthews correlation coefficient (the threshold is 50 GDT_TS)
AUC: The area under the ROC curve
Loss: The loss in quality between the best available model and the predicted best model
CASP11: 11th community wide experiment on the critical assessment of techniques for protein structure prediction
CASP12: 12th community wide experiment on the critical assessment of techniques for protein structure prediction
